# Supplementary figures and images for: Respiratory viruses activate autophagy via the IFN-STAT1/STAT5B-SOCS1 axis
Source: PLoS Pathog. 2026 Jul 15;22(7):e1014414. doi: 10.1371/journal.ppat.1014414 (PMC13395443; doi:10.1371/journal.ppat.1014414)

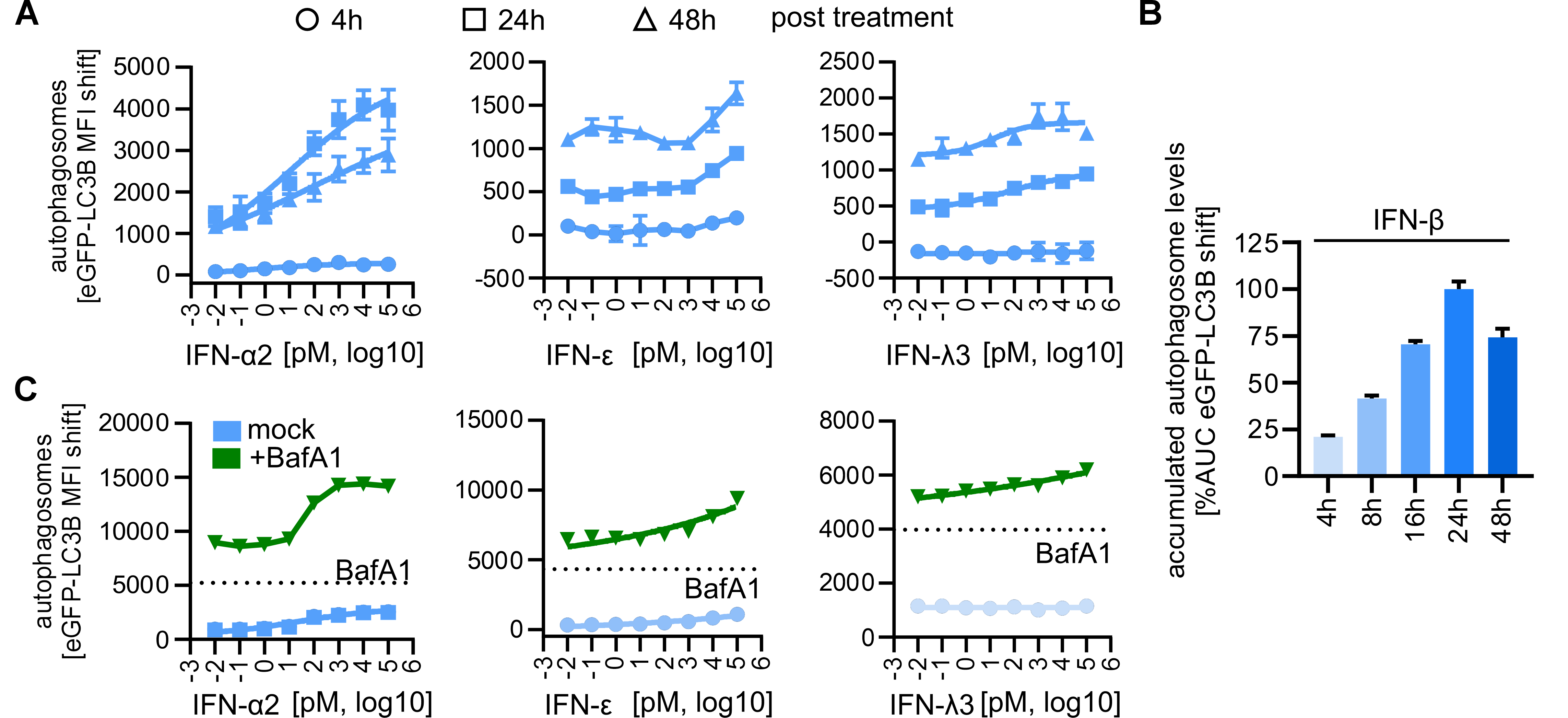

Supplement: S1 Fig — A, Quantification of autophagosome levels by flow cytometry in HeLa autophagy reporter cells (HeLa GL) 4, 24 and 48 h after treatment with increasing concentrations (0.01 pM – 100 nM) of indicated IFNs. n = 4 ± SD. B, Area under the curve analysis of accumulated autophagy levels over time normalized by highest induction (AUC) of data in Fig 1A and additionally 8 and 16 h after treatment with IFN-β. n = 4–8 ± SEM. C, Quantification of autophagosome levels by flow cytometry in HeLa autophagy reporter cells (HeLa GL) 24 after treatment with increasing concentrations (0.01 pM – 100 nM) of indicated IFNs in presence of 625 nM of the autophagy flux inhibitor Bafilomycin A1. Dotted line, Bafilomycin A1 only treatment. n = 4 ± SD. (TIF) [file ppat.1014414.s001.tif]

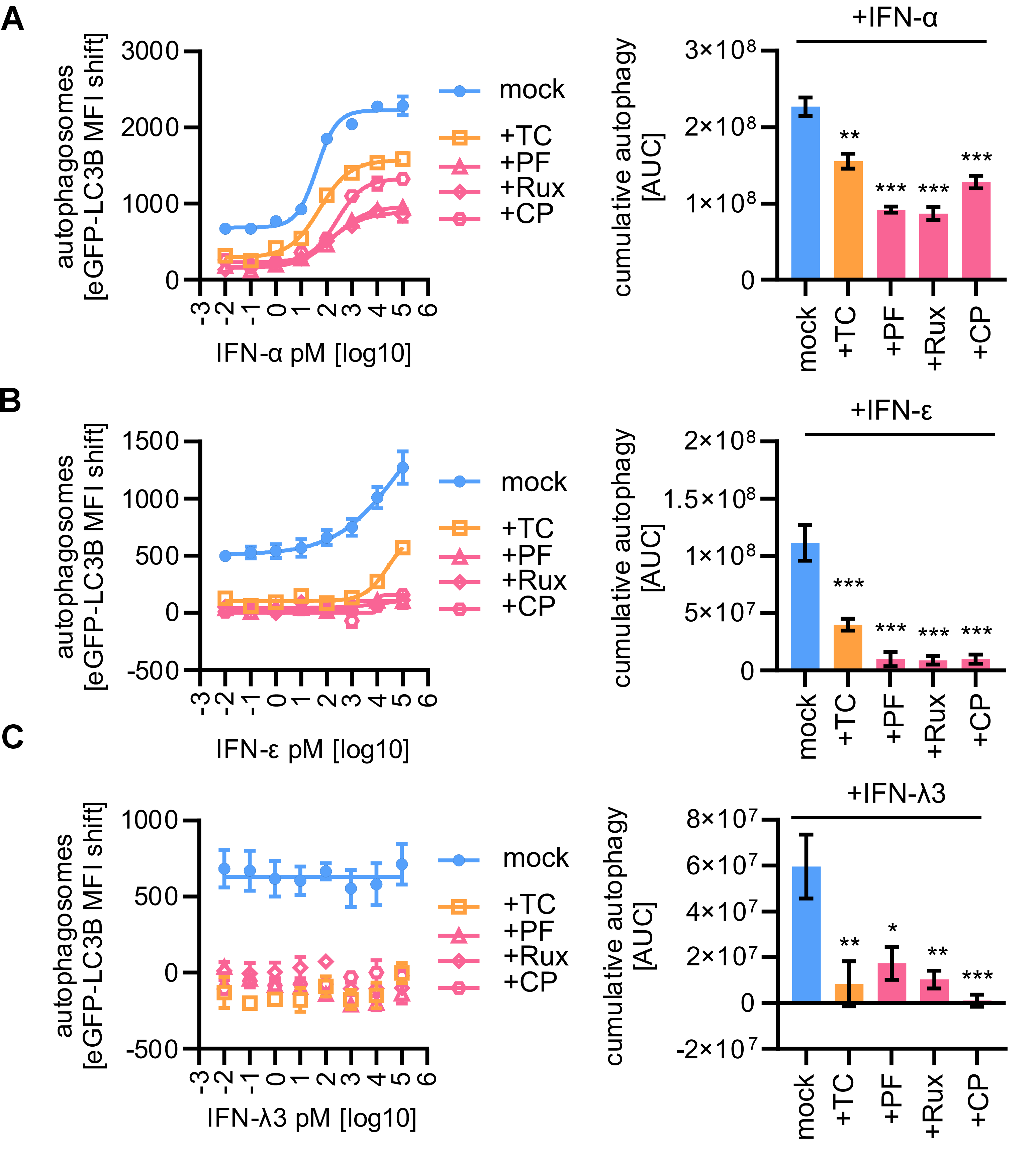

Supplement: S2 Fig — A-C, Quantification of autophagosome levels by flow cytometry in HeLa autophagy reporter cells (HeLa GL) 24 h after treatment with increasing concentrations (0.01 pM – 100 nM) of IFN-α (A), IFN-ε (B) or IFN-λ3 (C). Treated as indicated with TC JL 37 (TC, 100 nM), PF06551600 malonate (PF, Ritlecitinib, 100 µM), Ruxolitinib (Rux, 100 nM), or CP 690550 citrate (CP, Tofacitinib citrate, 100 nM), n = 4 ± SEM (left panels). Area under the curve (AUC) analysis of the data in (A-C), (right panels). Ordinary one-way ANOVA with Dunnett’s multiple comparisons test. * p < 0.05, ** p < 0.01, *** p < 0.001. (TIF) [file ppat.1014414.s002.tif]

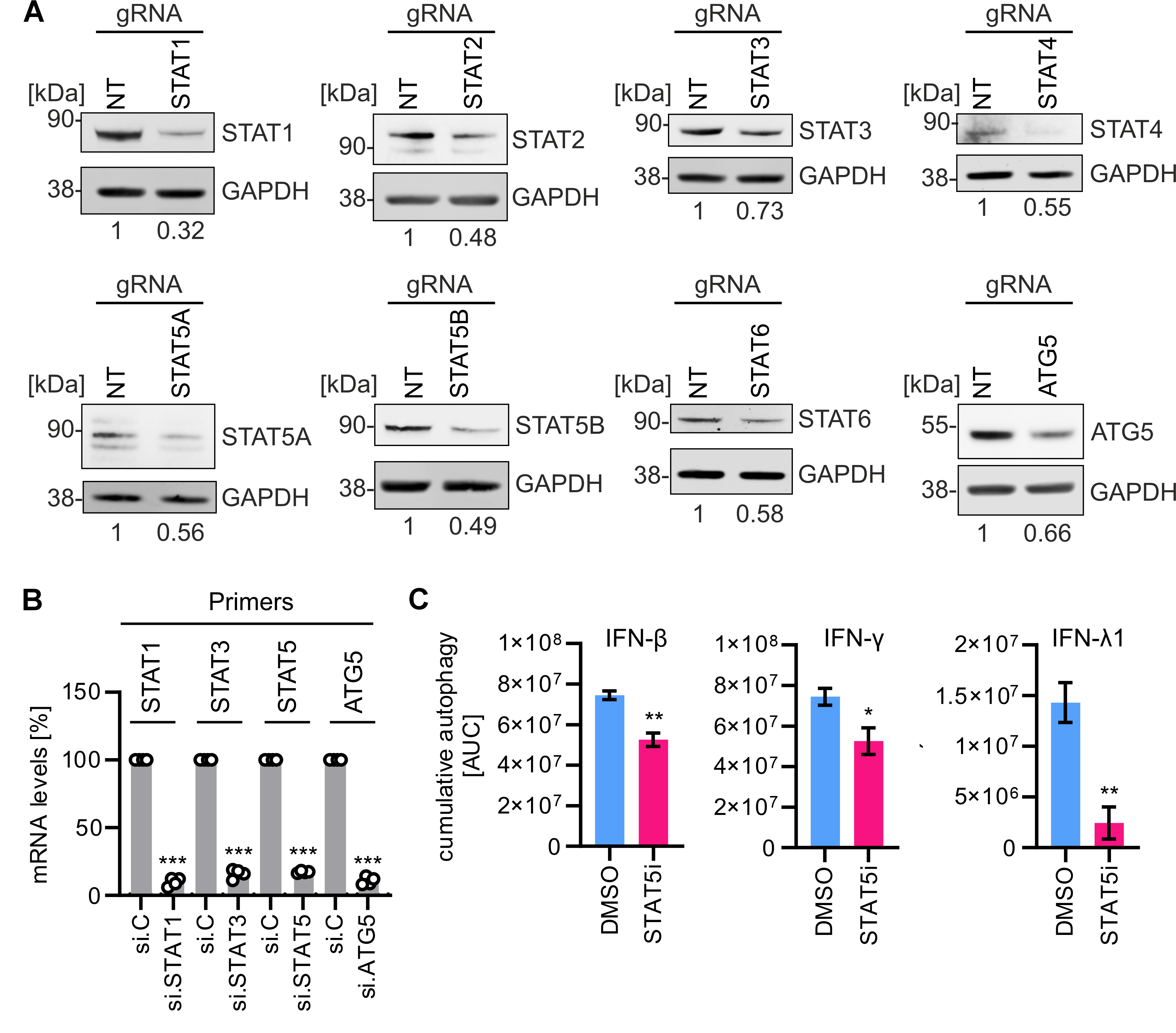

Supplement: S3 Fig — A, Representative immunoblots of whole cell lysates of HEKT293T-GL cells transfected as in Fig 3A. Individual STATs were detected with indicated antibodies. KD efficiency is indicated below the blots. Uncropped blots in S1 Raw Gel. B, RT-qRT-PCR analysis of siRNA-mediated knockdown of mRNA expression of data in Fig 3B. n = 4 ± SEM Student’s t-test with Welch’s correction. *** p < 0.001. C, Area under the curve (AUC) analysis of the data in Fig 3E and 3F. n = 4 ± SEM Student’s t-test with Welch’s correction. ** p < 0.01. (TIF) [file ppat.1014414.s003.tif]

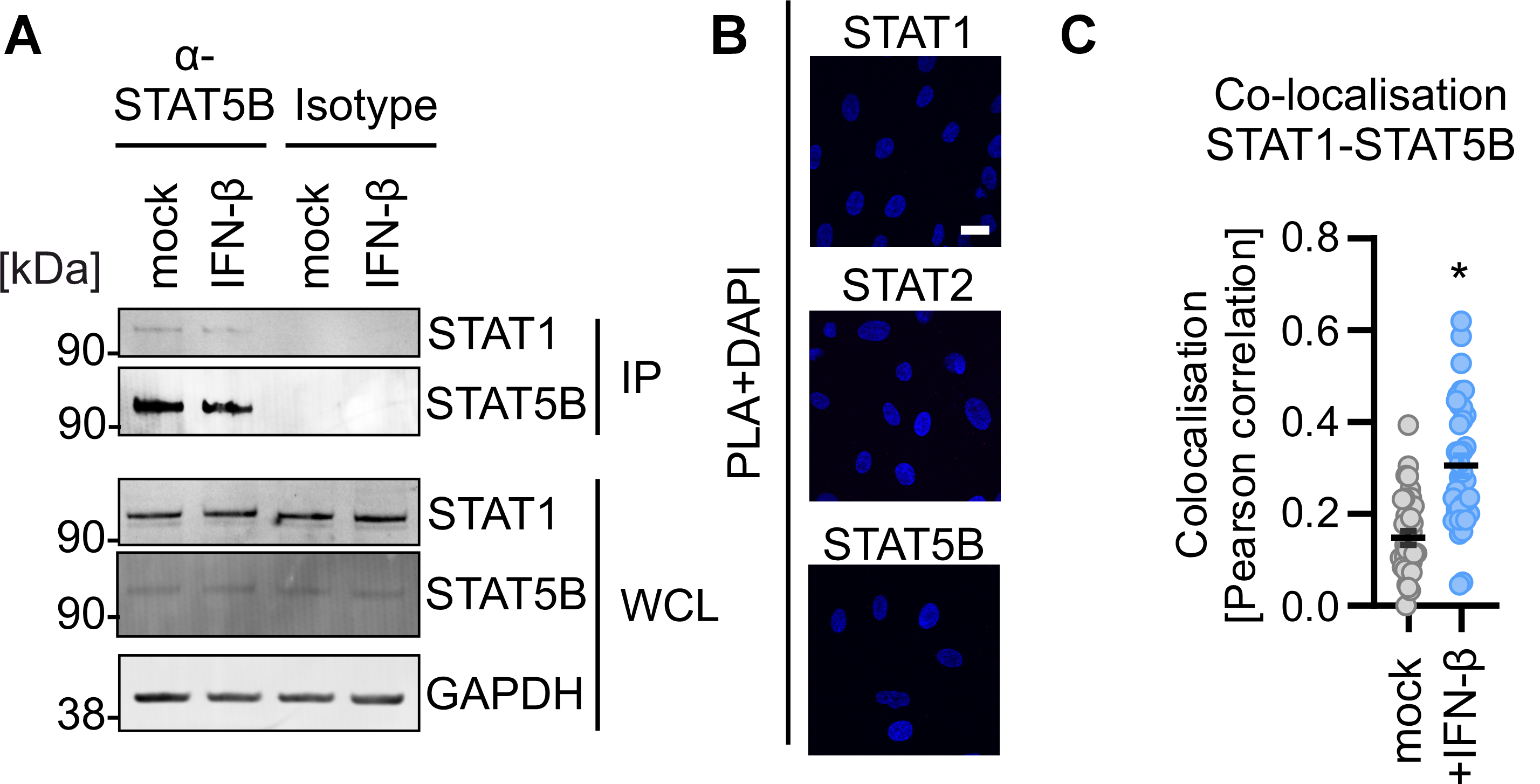

Supplement: S4 Fig — A, Exemplary Immunoblot of the co-immunoprecipitation of endogenous STAT1 and STAT5B in NHLF cells treated with 1 nM IFN-β for 1 h or left untreated. Uncropped blots in S1 Raw Gel. B, Exemplary confocal immunofluorescence images of single antibody controls for PLA in HDF hTERT cells of data in Fig 4C. PLA signal red. DAPI, nuclei (blue). (scale bar = 10 µm). C, Pearson Correlation of STAT1 and STAT5B signal of immunofluorescence data in Fig 4D. n = 37–38 ± SEM Student’s t-test with Welch’s correction. *** p < 0.0001. (TIF) [file ppat.1014414.s004.tif]

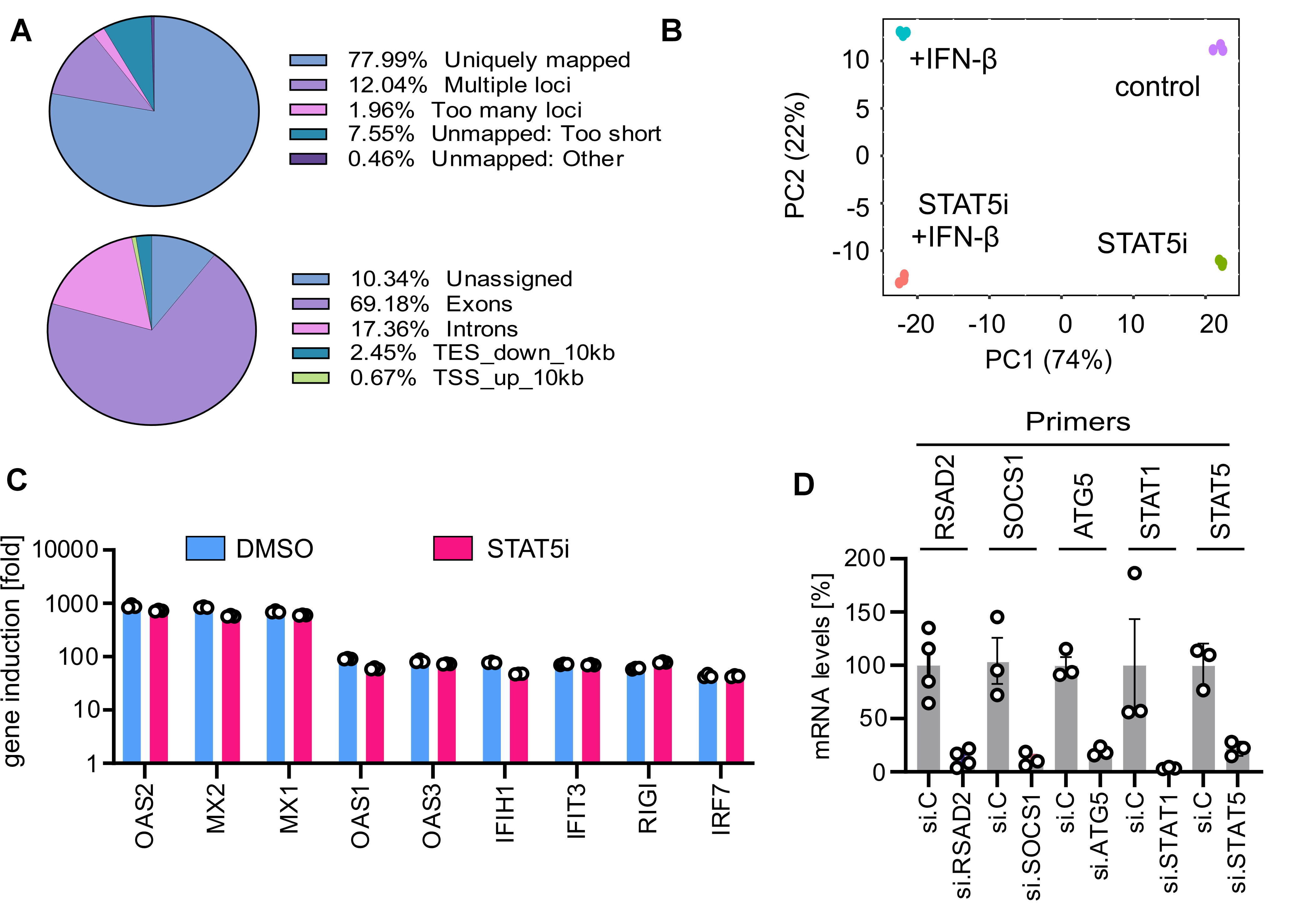

Supplement: S5 Fig — A, Percentage of mapped and unmapped reads for all samples n = 12 (top panel). Percentage of read tags to genome features for all samples. n = 12 (bottom panel). B, Principal Component Analysis of differential expression data, the individual samples are separated (violet control, cyan IFN-β, green STAT5 Inhibitor, orange IFN-β + STAT5 Inhibitor). n = 3. C, Fold changes of selected ISGs from the data in Fig 5A. n = 3 ± SEM. D, RT-qRT-PCR analysis of siRNA-mediated knockdown of mRNA expression of data in Fig 5F. n = 3–4 ± SEM. (TIF) [file ppat.1014414.s005.tif]

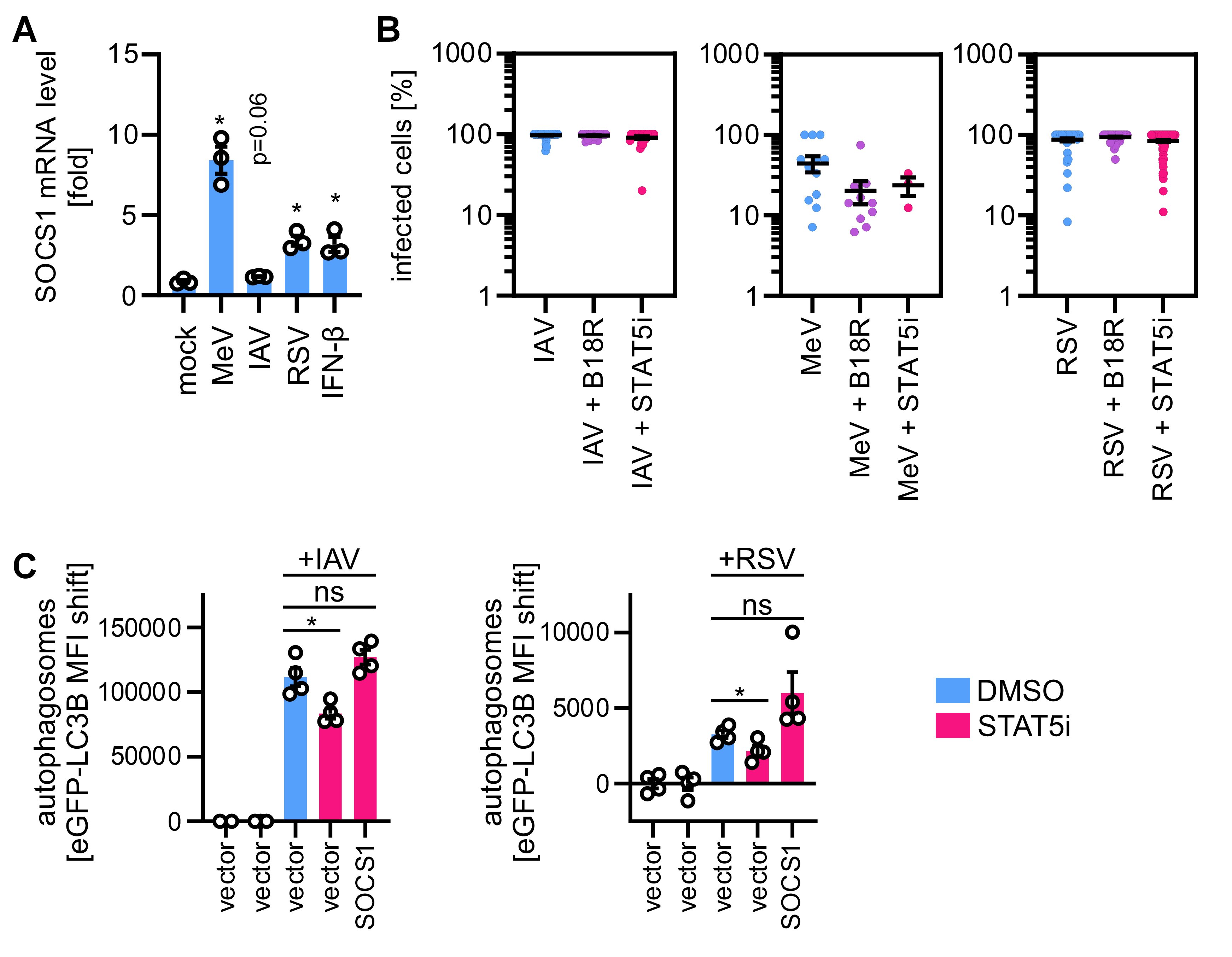

Supplement: S6 Fig — A, Quantification of SOCS1 mRNA levels by RT-qRT-PCR in NHLF cells 24 h post infection with Measles Virus (MeV, MOI 1.25), Respiratory Syncytial Virus (RSV, MOI 1.25), or Influenza A Virus (IAV, MOI 1.25). IFN-β (1 nM, 24h) was used as a control. n = 3–4 ± SEM. B, Percentage of infected cells per tile of immunofluorescence data in Fig 6D. n = 3–125 ± SEM. C, Autophagosome levels as quantified by flow cytometry in HEK293T GL cells infected with either IAV (MOI 1.25, left panel) 24 h post infection or RSV (MOI 1.25, right panel) 48 h post infection. Cells were either vector or SOCS1 transfected and treated with STAT5i as indicated (100 µM). n = 4 ± SEM. Student’s t-test with Welch’s correction. * p < 0.05; ns, not significant. (TIF) [file ppat.1014414.s006.tif]
