# Supplementary material for: Respiratory viruses activate autophagy via the IFN-STAT1/STAT5B-SOCS1 axis
Source: PLoS Pathog. 2026 Jul 15;22(7):e1014414. doi: 10.1371/journal.ppat.1014414 (PMC13395443; doi:10.1371/journal.ppat.1014414)
Supplement: Attachment — Submitted filename: 2026-06-02-Point-by-Point Response_.pdf [file ppat.1014414.s010.pdf]

Response to reviewers PPATHOGENS-D-25-02708
"Respiratory viruses activate autophagy via the IFN-STAT1/STAT5B-SOCS1 axis"
in blue

Reviewer #1: Autophagy, the process of recycling of intracellular components and pathogen clearance, plays an important role in both homeostasis and innate immune responses. In this manuscript, the authors are establishing a link between interferon induction of STAT1/STAT5b that mediates an alteration of SOCS1 resulting in induction of autophagy. This is critical as certain respiratory viruses exploit the autophagy pathway for their own replication. The outstanding question was how are interferon responses that induce autophagy are distinct from antiviral responses. The authors provide evidence that different types of interferon treatment can induce autophagy which is dependent on the Janus Kinase proteins as well as certain STAT proteins, specifically STAT1 and STAT5B which work together to induce autophagy. Importantly, inhibition of STAT1 and/or STAT5B thereby limiting autophagy reduces the capacity of a select group of respiratory viruses to replicate.

While well written, there remains significant concerns about the novelty of the study and the lack of controls which undermines the conclusions that can be derived from the results. In other cases the negative and positive controls used provide ambiguous results. Based on the list of concerns below, significant modification of the manuscript would be necessary.

Concerns are raised about the novelty of the study as it is well established that interferon treatment induces the autophagy pathway with numerous publications on this topic. Much of the study is well trodden ground.

We thank the reviewer for the comments. While we agree, that it has already been shown that IFNs induce autophagy, the underlying molecular mechanism(s), range of IFNs that induce autophagy, as well as the role of this connection during viral infection remained unclear. In our manuscript, we show that autophagy induction via STAT5-SOCS1 signalling is a feature of all main types of IFN. Furthermore, we provide evidence that autophagy and anti-viral gene induction by IFNs can be dissected, i.e. inhibition of STAT5 signalling disturbs only autophagy. Finally, we added new data showing that SOCS1 is induced during viral infection and that overexpression of SOCS1 largely rescues virus-induced autophagy and replication that were inhibited by STAT5 targeting (new Figs S6A, S6C, 6F).

Specific concerns:
1) Figure 1a needs a non-infected control to show baseline levels of autophagy.

The data was normalized to the mock control so we previously did not show it. In the updated figures we included the values (updated Fig. 1A). The MeV panel was repeated to include the treatment-only control (B18R without infection).

2) On line 131 the authors suggest the B18R treatment returns autophagy levels to baseline which is not evident in Fig 1b especially with the RSV infection.

Thanks for pointing this out. The wording was adjusted to reflect the data more accurately (lines 131) and the baseline is now highlighted in the figure (updated Fig. 1C).

3) Fig 1H is too dark to show the differences enumerated.

The brightness of the images was enhanced and we added an inset to better visualize the p62 puncta (updated Fig. 1H).

4) Fig 4b lacks a negative control.

The negative controls for the IP in Fig. 4B are the vector transfected cells, as well as the individually overexpressed V5 constructs (lane 1 and 6,7).

5) The results shown in Fig4b suggests that the effects shown wiht expression of STAT1 and STAT5B are additive at best.

We assume that the reviewer refers to Fig. 4A, right panel. As the reviewer pointed out the effects are rather additive and we have toned down our wording to reflect this (lines 193-195). We assume that this is due to the endogenous STAT1 or STAT5B which is complementing the overexpressed STATs.

6) the positive control in Fig 4a, TRIM32, induces levels equal to individual STAT1 and STAT5B levels which the authors state only "slightly induce autophagasomes" (line 191).

We have chosen the wording ‘slightly’ as an increase is visible, however, not significant in a one-way ANOVA test. Only the combined overexpression of STAT1/STAT5B induces significantly higher levels of LC3B+ puncta. We have adjusted our wording (line 193-195). The quantification of this assay way revisited and more cells included in the analyses, resulting in the positive control (TRIM32) also being significant (updated Fig. 4A).

7) It is not clear when the transcriptome analysis was performed which is critical to establish direct vs epistatic effects on transcription.

As now specified (line 212, also in Materials and Methods section), the transcriptome analyses were performed 16h post treatment, to capture the initial transcription.

8) The negative control in Fig 5H (Torin-1) induced autophagy to levels equal to the positive control BafA1.

Torin-1 activates autophagy by inhibiting mTOR signalling. In Fig 5H, as expected, Torin-1 causes an increase of autophagosomes (LC3B-GFP membrane-associated signal). Thus, it behaves similar to Bafilomycin A1. In Fig. 1H, levels of p62 are analysed as a measure of autophagic flux. As expected, activation of de novo autophagy by Torin-1 decreases the levels of p62, whereas Bafilomycin A1 causes accumulation of p62 due to inhibiting autophagic flux. See also Klionsky et al, Autophagy, 2021.

9) Fig 6A needs a mock control (uninfected)

We now show the mock control and mock treated values in the revised Fig. 6A. Please note, that the MeV panel was repeated to include the treatment-only values.

Line 223 Fig 5F should read Fig 5E.

Corrected.

Fig 5D needs a legend attached.

Added.

Fig 6A needs units on the graphs.

Corrected.

Reviewer #2: This is a review of the manuscript by Hunszinger et al. entitled "Respiratory viruses activate autophagy via the IFN-STAT1/STAT5B-SOCS1 axis." In this manscuript the authors find that IFN produced during viral infection activates autophagy through a STAT1/STAT5B dependent pathway that results in the upregulation of SOCS1 which stimulates autophagy through an undefined mechanisms. Furthermore, the inhibition of STAT5 reduces expression of only a subset of ISGs potentially allowing for the inhibition of STAT5 dependent upregulation of SOCS1 and induction of autophagy without compromising many innate immune defenses. Overall this is an interesting manuscript with several intriguing observation however there are a few issues that need to be addressed and that limit enthusiasm.

We thank the reviewer for her/his positive assessment of our work.

Major issues:

1. The authors propose an axis induced during viral infection that encompasses IFN◊STAT1/STAT5B◊ SOCS1which then activates autophagy to promote viral replication. While data presented throughout the manuscript support different aspects of this axis the experiments in the Figure 6 do not show that during viral infection that SOCS1 is induced or that its reduction due to STAT5i is directly responsible for the increase in autophagy and increased viral replication. In figure 6A does the overexpression of SOCS1 during viral infection + STAT5i treatment rescue the decrease in autophagy seen with STAT5i? In Figure 6D/E does the overexpression of SOCS1 during viral infection + STAT5i rescue viral replication? This is particularly important since SOCS1 is not the only ISG that is upregulated in a STAT5B dependent manner.

Our new qPCR data shows that SOCS1 is induced during viral infection (new Fig. S6A). Further, we now show that overexpression of SOCS1 rescues infection-induced autophagy (new Fig. S6C). Finally, in primary human lung fibroblasts, overexpression of SOCS1 partially rescues the impact of STAT5 inhibition on viral growth (new Fig. 6F). Thus, it is likely that the antiviral impact of STAT5i is largely mediated by SOCS1-induced autophagy. However, formally, we cannot exclude that other functions of STAT5 inhibition may contribute. We now address this in our discussion (lines 311-317).

2. In addition, key data to support the IFN-STAT1/STAT5B- SOCS1 axis is presented in figure 5F. Knockdown efficiency was shown for figure 5F in supplemental data S5D. In this figure there is no expression of SOCS1 with scrRNA or with siRNA targeting SOCS1 making interpretation of this figure impossible. Is SOCS1 even expressed in these cells?

Thanks for pointing this out, the data was omitted by mistake and is now included. We detect SOCS1 mRNA and show an efficient knockdown (updated Fig. S5D).

3. In Figure 3A the authors used CRISPR/Cas9 to knockout specific STAT genes however there is no data presented in either figure 3 or in the supplementary data validating the specific deletion, making interpretation of Figure 3A impossible.

This is a valid point. We now show on the protein level that individual STAT protein expression is reduced, corresponding to the individual gRNA used (new Fig. S3A).

Minor issues:

1. In Figure 3B the authors used STAT inhibitors however there is limited information provided as to where these inhibitors were obtained from or there specificity. At a minimum references need to be provided for these inhibitors to allow for evaluation of their specificity.

We have added information on the specificity and the references to the results text. All inhibitors were chosen for their property to target individual STATs (line 180-185).

2. For Figure 5D, E and F there is no legend provided to explain what the blue bar vs the pink bar represents. I also did not find a description in the figure legend.

We have added the description now to the figure legends, the pink bars represent STAT5i treated samples.

3. Within the manuscript the description of Figure 5F (lines 222-223) does not make sense and needs to be corrected.

Done (lines 223-226).

4. In Figure 5F the authors use siRNA targeted different ISGs and evaluate their impact on the induction of autophagy by IFN-B. Why did inhibition of STAT5B have minimal effect as compared to other figures.

Thanks for this comment. In some experiments the impact of the siRNA pool was lower than that of the gRNA or inhibitor. Closer analyses of the individual targeting sequences of the siRNA pool, revealed that the siRNAs also target STAT5A. This likely resulting in a mixed KD of both STAT5A and B, thus a weaker KD of STAT5B. The gRNA used were specific for STAT5B, whereas the inhibitor was used at a high concentration. We have now corrected all mentions of the siRNA pool to targeting STAT5 (A and B).

5. Labelling of the y axis is missing in Figure 6A.

Corrected.

Reviewer #3: Really well put together investigation on the role of IFN-signaling in the induction of autophagy. Data is supportive of the conclusions of the study. While the role of IFN-signaling in autophagy induction is not novel, the most interesting aspect of the study is the demonstrating that STAT5 upregulation of autophagy supports MeV and RSV growth.

Thanks for the positive assessment of our study!

Some clarity could be provided on whether inhibition of STAT5 can have other impacts on the infected cell, other than blocking autophagy, that might impact MeV and RSV survival.

This is an interesting point. To address this, we have now included an experiment showing that overexpression of SOCS1 rescues infection induced autophagy inhibited by STAT5 inhibition (new Fig. S6C). In addition, SOCS1 overexpression boosts replication of RSV upon STAT5 inhibition (new Fig 6F.) These assays suggest that SOCS1 is majorly responsible for the impact of STAT5 inhibition. Nonetheless, we cannot fully exclude that inhibition of STAT5 may impact viral replication in other ways. To address it, we added a brief paragraph to the discussion (lines 311-317).
