# Supplementary material for: Respiratory viruses activate autophagy via the IFN-STAT1/STAT5B-SOCS1 axis
Source: PLoS Pathog. 2026 Jul 15;22(7):e1014414. doi: 10.1371/journal.ppat.1014414 (PMC13395443; doi:10.1371/journal.ppat.1014414)
Supplement: Peer review history

# Peer review: Decision Letter 0

**DOI:** 10.1371/journal.ppat.1014414.r001

**Authors:** Matthias Johannes Schnell, Kai Zheng

11 Mar 2026

PPATHOGENS-D-25-02708

Respiratory viruses activate autophagy via the IFN-STAT1/STAT5B-SOCS1 axis

PLOS Pathogens

Dear Dr. Sparrer,

Thank you for submitting your manuscript to PLOS Pathogens. After careful consideration, we feel that it has merit but does not fully meet PLOS Pathogens's publication criteria as it currently stands. Therefore, we invite you to submit a revised version of the manuscript that addresses the points raised during the review process.

Please submit your revised manuscript by May 10 2026 11:59PM. If you will need more time than this to complete your revisions, please reply to this message or contact the journal office at plospathogens@plos.org. When you're ready to submit your revision, log on to https://www.editorialmanager.com/ppathogens/ and select the 'Submissions Needing Revision' folder to locate your manuscript file.

Please include the following items when submitting your revised manuscript:

* A letter that responds to each point raised by the editor and reviewer(s). You should upload this letter as a separate file labeled 'Response to Reviewers'. This file does not need to include responses to any formatting updates and technical items listed in the 'Journal Requirements' section below.

* A marked-up copy of your manuscript that highlights changes made to the original version. You should upload this as a separate file labeled 'Revised Manuscript with Track Changes'.

* An unmarked version of your revised paper without tracked changes. You should upload this as a separate file labeled 'Manuscript'.

If you would like to make changes to your financial disclosure, competing interests statement, or data availability statement, please make these updates within the submission form at the time of resubmission. Guidelines for resubmitting your figure files are available below the reviewer comments at the end of this letter.

We look forward to receiving your revised manuscript.

Kind regards,

Kai Zheng

Academic Editor

PLOS Pathogens

Matthias Schnell

Section Editor

PLOS Pathogens

Sumita Bhaduri-McIntosh

Editor-in-Chief

PLOS Pathogens

orcid.org/0000-0003-2946-9497

Michael Malim

Editor-in-Chief

PLOS Pathogens

orcid.org/0000-0002-7699-2064

**Journal Requirements:**

If the reviewer comments include a recommendation to cite specific previously published works, please review and evaluate these publications to determine whether they are relevant and should be cited. There is no requirement to cite these works unless the editor has indicated otherwise.

1) Please ensure that the CRediT author contributions listed for every co-author are completed accurately and in full.

At this stage, the following Authors/Authors require contributions: Victoria Hunszinger, Helen Dürr, Zoé Engels, Helene Hoenigsperger, Lennart Koepke, Susanne Klute, Jana-Romana Fischer, Birgit Ott, Alexander Graf, Stefan Krebs, Helmut Blum, Maximilian Hirschenberger, Frank Kirchhoff, and Konstantin Sparrer. Please ensure that the full contributions of each author are acknowledged in the "Add/Edit/Remove Authors" section of our submission form.

The list of CRediT author contributions may be found here: https://journals.plos.org/plospathogens/s/authorship#loc-author-contributions

2) Please upload all main figures as separate Figure files in .tif or .eps format. For more information about how to convert and format your figure files please see our guidelines:

https://journals.plos.org/plospathogens/s/figures

3) Please amend your detailed Financial Disclosure statement. This is published with the article. It must therefore be completed in full sentences and contain the exact wording you wish to be published.

1) State what role the funders took in the study. If the funders had no role in your study, please state: "The funders had no role in study design, data collection and analysis, decision to publish, or preparation of the manuscript."

2) If any authors received a salary from any of your funders, please state which authors and which funders..

If you did not receive any funding for this study, please simply state: u201cThe authors received no specific funding for this work.u201d

4) Your current Financial Disclosure states, "Yes Please add funding details. This study was supported by the German ministry for education and research grant IMMUNOMOD-01KI2014 (K.S.), the Baden-Wuerttemberg Foundation grant AutophagyBoost (K.S.), and the German Research Foundation (CRC1279 (F.K., K.S.), SP 1600/13-1 (K.S.), SP 1600/9-1 (K.S.)). ↳ Please select the country of your main research funder (please select carefully as in some cases this is used in fee calculation). GERMANY - DE".

However, your funding information on the submission form indicates different order.

Please indicate by return email the full and correct funding information for your study and confirm the order in which funding contributions should appear. Please be sure to indicate whether the funders played any role in the study design, data collection and analysis, decision to publish, or preparation of the manuscript.

**Reviewers' Comments:**

Reviewer's Responses to Questions

**Part I - Summary**

Please use this section to discuss strengths/weaknesses of study, novelty/significance, general execution and scholarship.

Reviewer #1: Autophagy, the process of recycling of intracellular components and pathogen clearance, plays an important role in both homeostasis and innate immune responses. In this manuscript, the authors are establishing a link between interferon induction of STAT1/STAT5b that mediates an alteration of SOCS1 resulting in induction of autophagy. This is critical as certain respiratory viruses exploit the autophagy pathway for their own replication. The outstanding question was how are interferon responses that induce autophagy are distinct from antiviral responses. The authors provide evidence that different types of interferon treatment can induce autophagy which is dependent on the Janus Kinase proteins as well as certain STAT proteins, specifically STAT1 and STAT5B which work together to induce autophagy. Importantly, inhibition of STAT1 and/or STAT5B thereby limiting autophagy reduces the capacity of a select group of respiratory viruses to replicate.

While well written, there remains significant concerns about the novelty of the study and the lack of controls which undermines the conclusions that can be derived from the results. In other cases the negative and positive controls used provide ambiguous results. Based on the list of concerns below, significant modification of the manuscript would be necessary.

Reviewer #2: This is a review of the manuscript by Hunszinger et al. entitled “Respiratory viruses activate autophagy via the IFN-STAT1/STAT5B-SOCS1 axis.” In this manscuript the authors find that IFN produced during viral infection activates autophagy through a STAT1/STAT5B dependent pathway that results in the upregulation of SOCS1 which stimulates autophagy through an undefined mechanisms. Furthermore, the inhibition of STAT5 reduces expression of only a subset of ISGs potentially allowing for the inhibition of STAT5 dependent upregulation of SOCS1 and induction of autophagy without compromising many innate immune defenses. Overall this is an interesting manuscript with several intriguing observation however there are a few issues that need to be addressed and that limit enthusiasm.

Reviewer #3: Really well put together investigation on the role of IFN-signaling in the induction of autophagy. Data is supportive of the conclusions of the study. While the role of IFN-signaling in autophagy induction is not novel, the most interesting aspect of the study is the demonstrating that STAT5 upregulation of autophagy supports MeV and RSV growth.

**********

**Part II – Major Issues: Key Experiments Required for Acceptance**

Please use this section to detail the key new experiments or modifications of existing experiments that should be absolutely required to validate study conclusions.

Generally, there should be no more than 3 such required experiments or major modifications for a "Major Revision" recommendation. If more than 3 experiments are necessary to validate the study conclusions, then you are encouraged to recommend "Reject".

Reviewer #1: Concerns are raised about the novelty of the study as it is well established that interferon treatment induces the autophagy pathway with numerous publications on this topic. Much of the study is well trodden ground.

Specific concerns:

1) Figure 1a needs a non-infected control to show baseline levels of autophagy.

2) On line 131 the authors suggest the B18R treatment returns autophagy levels to baseline which is not evident in Fig 1b especially with the RSV infection.

3) Fig 1H is too dark to show the differences enumerated.

4) Fig 4b lacks a negative control.

5) The results shown in Fig4b suggests that the effects shown wiht expression of STAT1 and STAT5B are additive at best.

6) the positive control in Fig 4a, TRIM32, induces levels equal to individual STAT1 and STAT5B levels which the authors state only "slightly induce autophagasomes" (line 191).

7) It is not clear when the transcriptome analysis was performed which is critical to establish direct vs epistatic effects on transcription.

8) The negative control in Fig 5H (Torin-1) induced autophagy to levels equal to the positive control BafA1.

9) Fig 6A needs a mock control (uninfected)

Reviewer #2: Major issues:

1. The authors propose an axis induced during viral infection that encompasses IFNSTAT1/STAT5B SOCS1which then activates autophagy to promote viral replication. While data presented throughout the manuscript support different aspects of this axis the experiments in the Figure 6 do not show that during viral infection that SOCS1 is induced or that its reduction due to STAT5i is directly responsible for the increase in autophagy and increased viral replication. In figure 6A does the overexpression of SOCS1 during viral infection + STAT5i treatment rescue the decrease in autophagy seen with STAT5i? In Figure 6D/E does the overexpression of SOCS1 during viral infection + STAT5i rescue viral replication? This is particularly important since SOCS1 is not the only ISG that is upregulated in a STAT5B dependent manner.

2. In addition, key data to support the IFN-STAT1/STAT5B- SOCS1 axis is presented in figure 5F. Knockdown efficiency was shown for figure 5F in supplemental data S5D. In this figure there is no expression of SOCS1 with scrRNA or with siRNA targeting SOCS1 making interpretation of this figure impossible. Is SOCS1 even expressed in these cells?

3. In Figure 3A the authors used CRISPR/Cas9 to knockout specific STAT genes however there is no data presented in either figure 3 or in the supplementary data validating the specific deletion, making interpretation of Figure 3A impossible.

Reviewer #3: No major issues

**********

**Part III – Minor Issues: Editorial and Data Presentation Modifications**

Please use this section for editorial suggestions as well as relatively minor modifications of existing data that would enhance clarity.

Reviewer #1: Line 223 Fig 5F should read Fig 5E.

Fig 5D needs a legend attached

Fig 6A needs units on the graphs.

Reviewer #2: Minor issues:

1. In Figure 3B the authors used STAT inhibitors however there is limited information provided as to where these inhibitors were obtained from or there specificity. At a minimum references need to be provided for these inhibitors to allow for evaluation of their specificity.

2. For Figure 5D, E and F there is no legend provided to explain what the blue bar vs the pink bar represents. I also did not find a description in the figure legend.

3. Within the manuscript the description of Figure 5F (lines 222-223) does not make sense and needs to be corrected.

4. In Figure 5F the authors use siRNA targeted different ISGs and evaluate their impact on the induction of autophagy by IFN-B. Why did inhibition of STAT5B have minimal effect as compared to other figures.

5. Labelling of the y axis is missing in Figure 6A.

Reviewer #3: Some clarity could be provided on whether inhibition of STAT5 can have other impacts on the infected cell, other than blocking autophagy, that might impact MeV and RSV survival.

**********

PLOS authors have the option to publish the peer review history of their article (what does this mean?). If published, this will include your full peer review and any attached files.

If you choose “no”, your identity will remain anonymous but your review may still be made public.

**Do you want your identity to be public for this peer review?**  For information about this choice, including consent withdrawal, please see our Privacy Policy.

Reviewer #1: No

Reviewer #2: No

Reviewer #3: No

**Figure resubmission:**

-->While revising your submission, we strongly recommend that you use PLOS’s NAAS tool (https://ngplosjournals.pagemajik.ai/artanalysis) to test your figure files. NAAS can convert your figure files to the TIFF file type and meet basic requirements (such as print size, resolution), or provide you with a report on issues that do not meet our requirements and that NAAS cannot fix.

After uploading your figures to PLOS’s NAAS tool - https://ngplosjournals.pagemajik.ai/artanalysis, NAAS will process the files provided and display the results in the "Uploaded Files" section of the page as the processing is complete. If the uploaded figures meet our requirements (or NAAS is able to fix the files to meet our requirements), the figure will be marked as "fixed" above. If NAAS is unable to fix the files, a red "failed" label will appear above. When NAAS has confirmed that the figure files meet our requirements, please download the file via the download option, and include these NAAS processed figure files when submitting your revised manuscript.

**Reproducibility:**

To enhance the reproducibility of your results, we recommend that authors of applicable studies deposit laboratory protocols in protocols.io, where a protocol can be assigned its own identifier (DOI) such that it can be cited independently in the future. Additionally, PLOS ONE offers an option to publish peer-reviewed clinical study protocols. Read more information on sharing protocols at https://plos.org/protocols?utm_medium=editorial-email&utm_source=authorletters&utm_campaign=protocols

# Author comment: Author response to Decision Letter 1

**DOI:** 10.1371/journal.ppat.1014414.r002

4 Jun 2026

Attachment Submitted filename: 2026-06-02-Point-by-Point Response_.pdf

# Peer review: Decision Letter 1

**DOI:** 10.1371/journal.ppat.1014414.r003

**Authors:** Matthias Johannes Schnell, Kai Zheng

25 Jun 2026

Dear Dr. Sparrer,

We are pleased to inform you that your manuscript 'Respiratory viruses activate autophagy via the IFN-STAT1/STAT5B-SOCS1 axis' has been provisionally accepted for publication in PLOS Pathogens.

Before your manuscript can be formally accepted you will need to complete some formatting changes, which you will receive in a follow up email. A member of our team will be in touch with a set of requests.

Please note that your manuscript will not be scheduled for publication until you have made the required changes, so a swift response is appreciated.

IMPORTANT: The editorial review process is now complete. PLOS will only permit corrections to spelling, formatting or significant scientific errors from this point onwards. Requests for major changes, or any which affect the scientific understanding of your work, will cause delays to the publication date of your manuscript.

Should you, your institution's press office or the journal office choose to press release your paper, you will automatically be opted out of early publication. We ask that you notify us now if you or your institution is planning to press release the article. All press must be co-ordinated with PLOS.

Thank you again for supporting Open Access publishing; we are looking forward to publishing your work in PLOS Pathogens.

Best regards,

Kai Zheng

Academic Editor

PLOS Pathogens

Matthias Schnell

Section Editor

PLOS Pathogens

Sumita Bhaduri-McIntosh

Editor-in-Chief

PLOS Pathogens

orcid.org/0000-0003-2946-9497

Michael Malim

Editor-in-Chief

PLOS Pathogens

orcid.org/0000-0002-7699-2064

***********************************************************

Reviewer Comments (if any, and for reference):

Reviewer's Responses to Questions

**Part I - Summary**

Please use this section to discuss strengths/weaknesses of study, novelty/significance, general execution and scholarship.

Reviewer #1: The authors have done a nice job of modifying their original submission to accommodate the concerns raised by all three reviewers of the original submission. No further concerns remain.

Reviewer #2: This is a revision of the manuscript by Hunszinger et al. entitled “Respiratory

viruses activate autophagy via the IFN-STAT1/STAT5B-SOCS1 axis.” In this manuscript the

authors find that IFN produced during viral infection activates autophagy through a

STAT1/STAT5B dependent pathway that results in the upregulation of SOCS1 which

stimulates autophagy through an undefined mechanism. Furthermore, the inhibition of STAT5

reduces expression of only a subset of ISGs potentially allowing for the inhibition of STAT5

dependent upregulation of SOCS1 and induction of autophagy without compromising many

innate immune defenses. The authors have addressed my major concerns with the manuscript. Most importantly demonstrating that the overexpression of SOCS1 rescues infection-induced autophagy and rescues the impact of STAT5 inhibition on viral growth. Furthermore, they show that the antiviral impact of STAT5i is partially mediated by SOCS1-induced autophagy. Minor criticism is that the description of the data within the results section for Figure 6F should be modified to state is partially rescues the phenotype, as was done in the discussion. Other concerns/corrections have been addressed.

Reviewer #3: No concerns. The authors have responded appropriately to reviewer comments.

**********

**Part II – Major Issues: Key Experiments Required for Acceptance**

Please use this section to detail the key new experiments or modifications of existing experiments that should be absolutely required to validate study conclusions.

Generally, there should be no more than 3 such required experiments or major modifications for a "Major Revision" recommendation. If more than 3 experiments are necessary to validate the study conclusions, then you are encouraged to recommend "Reject".

Reviewer #1: None

Reviewer #2: None

Reviewer #3: (No Response)

**********

**Part III – Minor Issues: Editorial and Data Presentation Modifications**

Please use this section for editorial suggestions as well as relatively minor modifications of existing data that would enhance clarity.

Reviewer #1: None

Reviewer #2: See above in regards to description of Figure 6F.

Reviewer #3: (No Response)

**********

PLOS authors have the option to publish the peer review history of their article (what does this mean?). If published, this will include your full peer review and any attached files.

If you choose “no”, your identity will remain anonymous but your review may still be made public.

**Do you want your identity to be public for this peer review?**  For information about this choice, including consent withdrawal, please see our Privacy Policy.

Reviewer #1: No

Reviewer #2: No

Reviewer #3: No

# Editor report: Acceptance letter

**DOI:** 10.1371/journal.ppat.1014414.r004

**Authors:** Matthias Johannes Schnell, Kai Zheng

Dear Dr. Sparrer,

We are delighted to inform you that your manuscript, "Respiratory viruses activate autophagy via the IFN-STAT1/STAT5B-SOCS1 axis," has been formally accepted for publication in PLOS Pathogens.

We have now passed your article onto the PLOS Production Department who will complete the rest of the pre-publication process. All authors will receive a confirmation email upon publication.

The corresponding author will soon be receiving a typeset proof for review, to ensure errors have not been introduced during production. Please review the PDF proof of your manuscript carefully, as this is the last chance to correct any scientific or type-setting errors. Please note that major changes, or those which affect the scientific understanding of the work, will likely cause delays to the publication date of your manuscript. Note: Proofs for Front Matter articles (Pearls, Reviews, Opinions, etc...) are generated on a different schedule and may not be made available as quickly.

Soon after your final files are uploaded, the early version of your manuscript, if you opted to have an early version of your article, will be published online. The date of the early version will be your article's publication date. The final article will be published to the same URL, and all versions of the paper will be accessible to readers.

For Research Articles, you will receive an invoice from PLOS for your publication fee after your manuscript has reached the completed accept phase. If you receive an email requesting payment before acceptance or for any other service, this may be a phishing scheme. Learn how to identify phishing emails and protect your accounts at https://explore.plos.org/phishing.

Thank you again for supporting open-access publishing; we are looking forward to publishing your work in PLOS Pathogens.

Best regards,

Sumita Bhaduri-McIntosh

Editor-in-Chief

PLOS Pathogens

orcid.org/0000-0003-2946-9497

Michael Malim

Editor-in-Chief

PLOS Pathogens

orcid.org/0000-0002-7699-2064
